# Roles of interstitial cells of Cajal in regulating gastrointestinal motility: *in vitro versus in vivo* studies

**DOI:** 10.1111/j.1582-4934.2008.00352.x

**Published:** 2008-04-18

**Authors:** Jieyun Yin, Jiande DZ Chen

**Affiliations:** Division of Gastroenterology, Department of Medicine, University of Texas Medical BranchGalveston, TX, USA

**Keywords:** ICC, gastrointestinal motility, electrogastrography, neurotransmission, slow waves, emptying

## Abstract

The aim of this article is to provide a better understanding of the roles of interstitial cells of Cajal (ICC) in regulating gastrointestinal motility by reviewing *in vitro* and *in vivo* physiological motility studies. Based on the *in vitro* studies, ICC are proposed to have the following functions: to generate slow waves, to mediate neurotransmission between the enteric nerves and the gastrointestinal muscles and to act as mechanoreceptors. However, there is limited evidence available for these hypotheses from the *in vivo* motility studies. In this review, we first introduce the major subtypes of ICC and their established functions. Three Kit mutant mouse and rodent models are presented and the loss of ICC subtypes in these mutants is reviewed. The physiological motility findings from various in vitroand *in vivo* experiments are discussed to give a critical review on the roles of ICC in generating slow waves, regulating gastrointestinal motility, mediating neural transmission and serving as mechanoreceptors. It is concluded that the role of ICC as pacemakers may be well established, but other cells may also be involved in the generation of slow waves; the theory that ICC are mediators of neurotransmission is challenged by the majority of the *in vivo* motility studies; the hypothesis that ICC are mechanoreceptors has not found supportive evidence from the *in vivo* studies yet. More studies are needed to explain discrepancies in motility findings between the *in vitro* and *in vivo* experiments.

- Subtypes and functions of ICC in the gut
  - Subtypes of ICC
  - ICC along the gut
  - Functions of ICC
- Mutant animal models used in *in vitro* and *in vivo* studies
- Roles of ICC in generating slow waves
  - Gastrointestinal slow waves and their clinical
  - significance
  - *In vitro* studies
  - *In vivo* studies
- Roles of ICC in regulating gastrointestinal motility
  - Gastrointestinal motility and roles of slow waves
  - Roles of ICC in regulating peristaltic contractions
- Roles of ICC in mediating neural transmission
  - *In vitro* studies
    - LOS and pylorus sphincter
    - Stomach
    - Small intestine
    - Colon
  - *In vivo* studies
    - Lower oesophageal sphincter
    - Stomach
    - Colon
    - Anal sphincter
- Roles of ICC as mechanoreceptors
- Conclusions

## Subtypes and functions of ICC in the gut

### Subtypes of ICC

The interstitial cells of Cajal (ICC) were first characterized by Cajal [1] and are now known to play an important role in gastrointestinal motility [2–5]. According to the location in the gut wall, ICC can be classified into following major subtypes: ICC-MY (ICC in the myen-teric plexus, also called ICC-AP or ICC-MP), ICC-IM (ICC within the circular and longitudinal layers of muscle), ICC-DMP (ICC in the deep muscular plexus) and ICC-SMP (ICC in the submuscular plexus).

### ICC along the gut

In the oesophagus, ICC are in the smooth muscle of the oesophagus and within the lower oesophageal sphincter (LOS). The ICC are of the ICC-IM subtype [6, 7]. In the stomach, the ICC are more densely located in the corpus and antrum than in the fundus. In the antrum, both ICC-MY and ICC-IM are present, whereas in the fun-dus, only ICC-IM are found [8–10]. Although there are conflicting reports, in general, the distribution of ICC-MY in the stomach is in agreement with the *in vivo* electrophysiological recording of slow waves responsible for gastric contractions. In the pylorus, there are ICC-IM [11, 12]. In the small intestine, there are ICC-MY, ICC-IM and ICC-DMP [13, 14]. Compared to the small intestine, there are less ICC in the colon; the ICC in the colon include subtypes of ICC-MY, ICC-IM and ICC-SMP [15, 16]. In the anorectal region, spindle-shaped ICC are present in both muscle layers, parallel to the smooth muscle cells. ICC are abundant, surrounding the myenteric ganglia. ICC at the submuscular plexus are less dense [17–20].

### Functions of ICC

Based on *in vitro* studies, ICC are theorized to have the following functions: (1) to pace the slow waves and regulate slow wave propagation. The involved subtypes of ICC for these functions are ICC-MY in the stomach and small intestine, and ICC-SMP in the colon [21–25]. (2) To mediate enteric neural signals to the smooth muscles. ICC-IM are considered to have this function [16, 26–28]. (3) To act as mechanosensors [29]. However, some of the above theories have been put into question by a number of *in vivo* studies that are reviewed in this article. The discrepancies between the *in vitro* and *in vivo* studies may suggest that the available mutant mice or rats have complicated physiologies than are usually assumed [30].

## Mutant animal models used in *in vitro* and *in vivo* studies

Three mutant models, W/W^v^ mice [31–33], Sl/Sl^d^, mice [34, 35], Ws/Ws rats [28, 36–41] have been used to investigate the roles of ICC in the regulation of gastrointestinal motility. Specific subtypes of ICC are obliterated, reduced in numbers or damaged at different locations in the gastrointestinal tract. Accordingly, these models provide unique opportunities for the investigation of the roles of various subtypes of ICC in different organs of the gut.

W/W^v^ mutants have been most frequently used in the investigation of the roles of ICC in regulating gastrointestinal motility. In the W/W^v^ mouse, there is a loss of ICC-IM in the LOS [42], an almost absence of ICC-IM in the stomach [43, 44], a loss of ICC-IM in the pylorus sphincter [42], an almost complete loss of ICC-MY in the small intestine [43, 44], a loss of ICC-MY in the middle and distal colon [45] and an absence of ICC-MY in the internal anal sphincter [17].

Similar to W/W^v^ mice, there is an almost complete loss of ICC-MY in the small intestine of Sl/Sl^d^ mice [34, 35]. The ICC-DMP in the small intestine was, however, found to be normal. It appears that the characteristics of ICC in Sl/Sl^d^ mice are similar to those of W/W^v^ mice, but few studies have been performed with the Sl/Sl^d^ mouse to investigate the roles of ICC in regulating gastrointestinal motility.

The Ws/Ws rat is also frequently used in the ICC studies. Since the rat is larger in size, certain *in vivo* studies are more feasible in the Ws/Ws rat than the W/W^v^ mouse [28, 36–41]. ICC-IM were found to be absent in the LOS [36], the antrum [37] and the pylorus [38]. The ICC-MY are present in the stomach [37]. In the small intestine, ICC-MY were reported to be absent, but ICC-DMP were present [39]. The density of Kit-positive cells in the DMP of Ws/Ws rats was similar to those in wild-type rats. ICC-DMP in the rat of both wild-type and Ws/Ws mutants were similar in structure to ICC-DMP of the mouse. In the colon, the loss of Kit-positive cells (all ICC subtypes) was found to be more than 90% in the middle and distal parts of the colon; the loss of ICC-SMP was almost complete and the loss of ICC-MY was about 50% in the proximal colon [28]. That is, the two most important subtypes of ICC in the colon, ICC-DMP and ICC-IM, are virtually completely absent.

In general, the loss of ICC subtypes in various locations along the gastrointestinal tract is comparable among these three mutant models of rats and mice.

## Roles of ICC in generating slow waves

### Gastrointestinal slow waves and their clinical significance

**Fig. 1:**
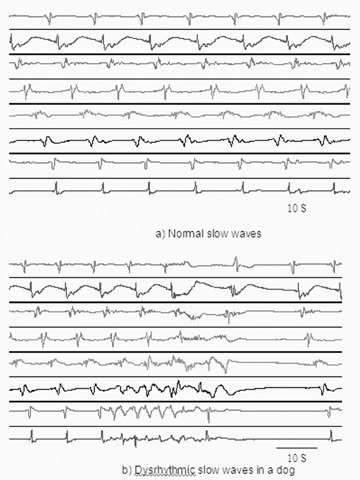
*In vivo* serosal recordings of normal gastric slow waves in dogs. The top tracing was from the electrodes 16 cm above the pylorus. The bottom tracing was from the electrodes 2 cm above the pylorus. The distance between the two channels was 2 cm. (**A**) Regular gastric slow waves and their distal propagations were observed. (**B**) Regular gastric slow waves and their distal propagations were observed at the beginning, and then, an ectopic pacemaker was present in the distal stomach, generating retrogradely propagated slow waves at a higher frequency.

In most of the clinical studies investigating the role of slow waves in controlling gastrointestinal motility, the slow waves are recorded using bipolar electrodes attached to the serosa or mucosa of the stomach or the abdominal skin [46]. The myoelectrical activity of the gut is composed of slow waves and spikes [47, 48]. According to *in vivo* serosal recordings, the fundus is believed to be electrically quiescent (without slow waves) and the corpus is where the pacemaker is located. The gastric slow wave propagates distally with an increased amplitude and velocity. The frequency of the slow wave is 3 cycles/min. (cpm) in human beings and 5 cpm in dogs. Figure 1 shows typical gastric slow waves measured from eight pairs of serosal electrodes along the greater curvature in a dog. Normal dis-tally propagated slow waves are present in the top panels (A).

According to the *in vivo* serosal recordings, the small intestinal slow waves originate from a region in the proximal 1 cm of the duodenum and propagate as an annular wavefront in an aborad direction [49]. It determines the frequency and the direction of propagation of intestinal contractions. In the dog, the proximal 10–30% of the small intestine (30–115 cm of the duodenum and jejunum) maintains the same slow wave frequency, 18–20 cpm, in a region called the ‘frequency plateau’[50]. Aborad to this point, there is a diminishing slow wave frequency gradient along the small bowel to a rate of 14 cpm in the distal ileum [50, 51]. In human beings, slow waves in the duodenum and proximal jejunum occur at about 12 cpm, with an aborad gradient to about 9 cpm in the terminal ileum [52, 53]. Whether a proximal plateau of identical frequencies is present in the human duodenum and proximal jejunum has not been clearly shown [52]. Transection and reanas-tomosis of the small bowel decrease the slow wave frequency in the distal segment in both dogs [54] and man [55]. In addition, at least in dogs, the propagation of slow waves in the distal segment becomes abnormal, with a high percentage of these slow waves propagating in an orad rather than an aborad direction [56].

The colonic slow waves in *in vivo* studies are not well characterized and have multiple frequencies without a dominant rhyth-micity. In a human study with bipolar serosal electrodes, it was reported that the ascending colon had a low level of signal that showed the simultaneous presence of variable and multiple frequency components in each of the two frequency ranges of 2–9 cpm and 9–13 cpm [57]. The slow wave in the transverse colon was characterized mostly by a single, stable frequency in the range of 9–13 cpm, with the presence of single or multiple frequencies in the range of 2–9 cpm. The means of slow wave frequencies were about 4 cpm and 11 cpm in the descending and sigmoid colon, respectively.

**Fig. 2:**
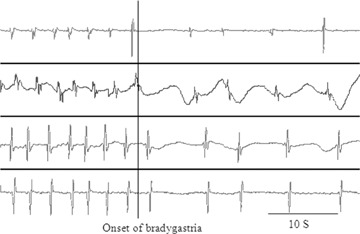
*In vivo* serosal recordings of normal and bradygastric slow waves in a dog. The top tracing was from the electrodes 16 cm above the pylorus. The bottom tracing was from the electrodes 2 cm above the pylorus. The distance between the two channels was 4 cm. Regular gastric slow waves and their distal propagations were observed at the beginning, followed with bradygastrial slow waves.

Slow wave dysrhythmias have been frequently reported in the stomach, but rarely in the small intestine and colon. The gastric slow waves and dysrhythmias can be detected using non-invasive methods of electrogastrography [46], whereas the measurement of intestinal and colonic slow waves in clinical settings is difficult since there is no non-invasive method. Gastric dysrhythmia includes bradygastria (slow wave frequency lower than normal), tachygastria (slow wave frequency higher than normal) and arrhythmia (no rhythmic slow waves). Numerous studies have shown that gastric dysrhythmia is associated with gastric motor disorders and/or gastrointestinal symptoms [46, 58–60]. A recent study in our laboratory has revealed that tachygastria is ectopic and of an antral origin [61]. In more than 80% of cases, tachygas-tria is located in the antrum and propagates retrogradely towards the pacemaker area of the proximal stomach. It may completely override normal distally propagating slow waves. However, most commonly, it does not completely override the normal gastric slow waves. In this case, there are two different slow wave activities: normal slow waves in the proximal stomach and tachygastrial slow waves in the distal stomach. A typical example is presented in Figure 1B: normal gastric slow waves propagating from the proximal stomach to the distal stomach were noted at the beginning of the tracing, and then, an ectopic pacemaker was present in the distal stomach firing slow waves at a higher frequency and propagating them orally to the proximal stomach. Close to the end of the tracings, the ectopic tachygastrial slow waves were overridden by the normal distally propagated slow waves. Tachygastria or tachyarrhythmia is associated with the absence or impairment of gastric contractions [60] and symptoms of nausea and vomiting [62, 63]. Unlike tachygastria, bradygastria is not ectopic and reflects purely a reduction in the frequency of normal pacemaking activity. That is, the entire stomach has one single frequency when bradygastria occurs [61]. Figure 2 illustrates a typical example: gastric slow waves were of a reduced frequency, but originated and propagated distally from the proximal stomach. It is seen that bradygastria originates in the corpus and propagates distally towards the pylorus. The gastric contractions may or may not be impaired with bradygastria [60].

Little is known about the pathophysiology of the intestinal slow waves. A few studies reported intestinal slow wave dysrhythmia in clinical settings such as nausea and vomiting, intestinal pseudo-obstruction and intestinal ischemia [62, 64–68]. The abnormalities in the intestinal slow waves include dysrhythmia, reduced frequency and uncoordinated slow waves along the intestine and are associated with impaired intestinal contractions.

Nothing has been reported on the pathophysiology of the *in vivo* colonic slow waves, mainly attributed to the following factors: (1) the slow waves in the colon are not well characterized, and there is no means of differentiating dysrhythmic slow waves from normal slow waves in the colon and (2) there is a lack of *in vivo* measurement methods for the colonic slow waves that can be applied in clinical settings.

### *In vitro* studies

A large number of *in vitro* studies have demonstrated the role of ICC as pacemakers with the evidence that can be summarized as follows: (1) ICC generate slow waves, (2) in the mutant animals where the ICC are absent, there are no slow waves and (3) in a specially prepared gastric tissue where ICC are obliterated by neutralizing antibody to Kit (activated Cdc42-associated kinase-2; ACK2), slow waves are absent [69]. A few studies from the groups of Huizinga and Ward performed with cultured ICC provided direct and strong evidence that the generation of pacemaker activity is an intrinsic property of the ICC [21, 70, 71]. Recordings from the intestinal muscles of the W/W^v^ mice in which ICC-MY are almost completely absent showed a complete loss of slow wave activity [72, 73]. Similar results were obtained from the muscles of Sl/Sl mice [34]. In order to investigate the role of ICC-MY in the generation of gastric slow waves, strips of gastric muscle tissues were incubated with the ACK2 for 31–50 days. This procedure obliterated all ICC, including ICC-MY, and abolished slow waves in the circular muscle cells [69]. Moreover, in the absence of ICC-MY, electrical field stimulation at a pulse width of about 122 msec. was not able to phase advance or pace gastric slow waves. These findings suggest that ICC-MY are pacemaker cells, and slow waves cannot be paced without ICC-MY.

### *In vivo* studies

**Fig. 3:**
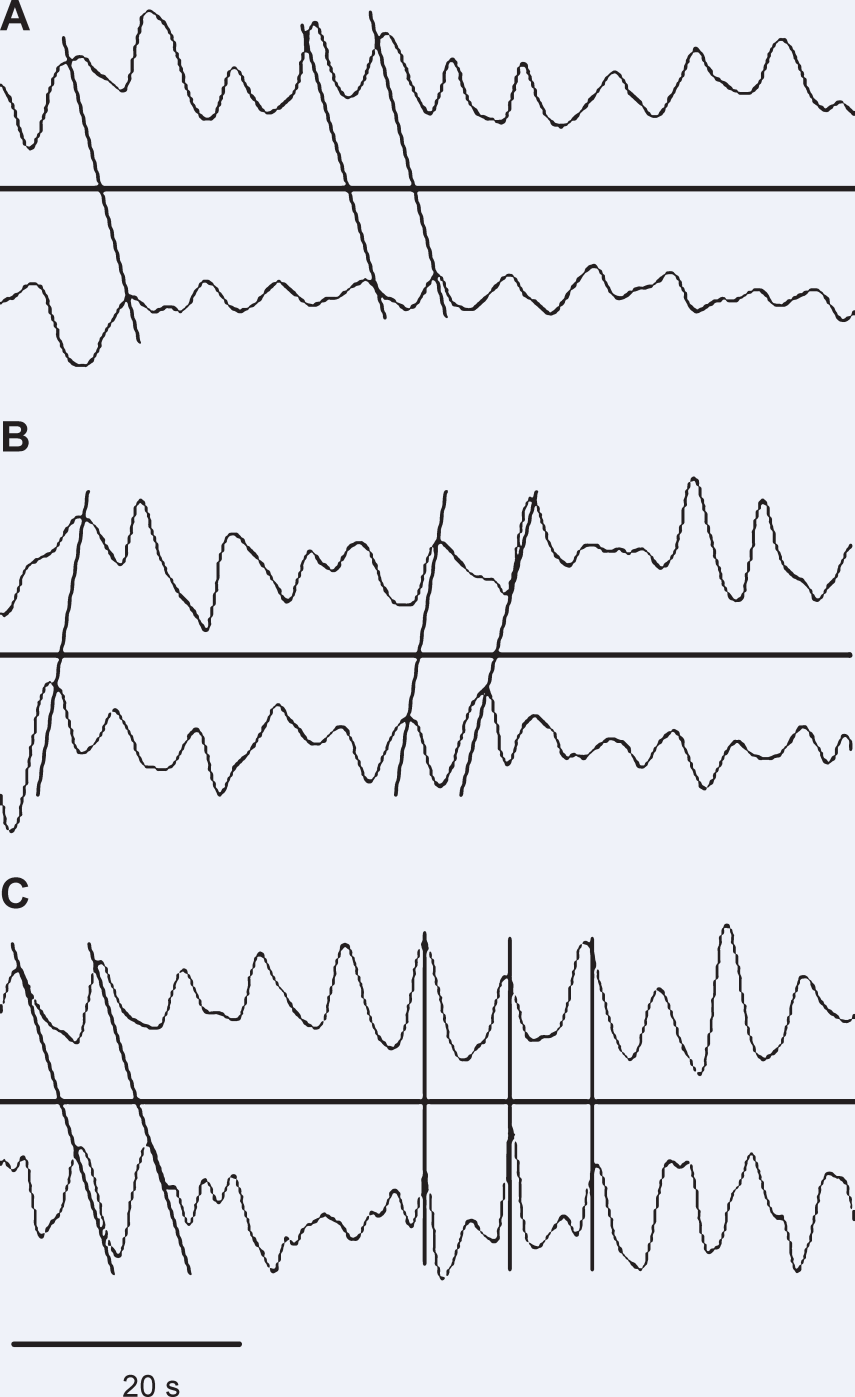
A 1-min. recording of intestinal slow waves in a conscious W/W^v^ mouse. The electrical signals were recorded from two pairs of electrodes placed on the serosal surface of the duodenum at an interval of 5 cm. Top channel in each panel was from the proximal pair of electrodes, whereas the bottom channel was from the distal pair of electrodes. (**A**) Antegrade propagation. (**B**) Retrograde propagation. (**C**) Antegrade propagation followed by an absence of propagation.

Conflicting results have been reported in a recent *in vivo* study suggesting that ICC may not be necessary for the generation of slow waves. In W/W^v^ mice (in which the pacemaking ICC, ICC-MY, are almost completely lost in the small intestine), gastrointestinal myoelectrical recordings were made from pairs of electrodes placed on the gastric and intestinal serosa. The slow waves were recorded in both anaesthetized and conscious W/W^v^ mice and they were not blocked by atropine or verapamil [74]. The *in vivo* slow waves recorded from the stomach of the W/W^v^ mice were identical to those from the stomach of the wild-type control mice. The *in vivo* slow waves from the small intestine of the W/W^v^ mice were, however, impaired compared to those recorded from the control mice, reflected as a decrease in the slow wave frequency (from 18.8 cpm to 10.7 cpm) and rhythmicity. Moreover, in comparison to the control rats, the W/W^v^ mice showed significantly reduced antegrade propagation and increased simultaneous and retrograde propagation. Various patterns of slow wave propagation are illustrated in Fig. 3. Similar findings were also reported in *in vitro* studies in W/W^v^ mice: the muscle strips taken from W/W^v^ mice in organ bath continued to generate slow waves and rhythmic phasic contractions, but both are more irregular in frequency and smaller in amplitude [72, 75–77]. Since the ICC-MY in the small intestine are almost completely lost in W/W^v^ mice, these findings suggest that ICC-MY may not be the sole pacemaker. Apparently, more studies, especially in vivostudies, are needed to explain the difference in the generation of slow waves between the *in vitro* studies and in vivorecordings.

Another *in vivo* study has suggested that ICC may not be needed for pacing slow waves [78]. In the W/W^v^ mice without ICC-MY in the small intestine, a complete entrainment of the intestinal slow waves was achieved in the same way as that in the control mice. The required stimulation pulse width was about 50 msec. and was the same for both control and W/W^v^ mice [78]. The finding of the study demonstrates that exogenous pacing can be achieved with the absence of ICC-MY. Similar findings are also reported in *in vitro* studies [79, 80]: in the intestine of W/W^v^ mice, there is a pacing system in the longitudinal muscle that does not require ICC-MY, and the absence of a normal network of ICC-MY does not abolish regular pacing activity. However, these studies were done to measure the contractile activity without actually recording the electrical pacemaker activity.

## Roles of ICC in regulating gastrointestinal motility

### Gastrointestinal motility and roles of slow waves

Gastrointestinal motility is important in the transportation of ingested food and absorption of nutrients along the gut. Upon food intake, the gastric fundus relaxes to accommodate the ingested food. Peristalsis (distally propagated contractions) generates in the proximal antrum and propagates distally to the pylorus. Before the antral contractile front reaches, the pylorus relaxes or opens, and the gastric chyme expels out to the small intestine through the pylorus. In the small intestine, mixed contractile patterns (antegrade, simultaneous and retrograde) are present in the post-prandial state. These mixed patterns of contractions are necessary for the absorption of nutrients and transport of the chyme along the small intestine. The contractile patterns in the colon are more complex and have not been well defined. The only well-defined pattern of colonic contractions is the giant migrating contractions that are known to transport faeces from the proximal colon to the distal colon.

In addition to the rhythmic contractions or peristalsis mentioned above, the gut also generates and maintains tone. The tone or resting pressure of various sphincters along the gut also plays important roles in the transport of the ingested food along the gut. The gut sphincters include the LOS, pylorus and anal sphincter. A decrease in LOS may lead to gastro-oesophageal reflux, whereas an impaired relaxation of LOS during swallows is one of major causes of dysphagia. Impaired pyloric relaxation may lead to pyloric stenosis or delayed gastric emptying. In the anus, the weakness of the anal sphincter is related to faecal incontinence, and the failure in the relaxation of the anal sphincter is associated with outlet-obstructive constipation.

Although numerous studies have reported the absence of ICC in various patient populations [81–89], the roles of ICC or slow waves in regulating gastrointestinal motility should not be exaggerated. Slow waves are known to determine the time, frequency and propagation of contractions. However, they do notdirectly initiate effective gastrointestinal contractions. The binding of acetylcholine to muscarinic receptors during slow wave depolarization is essential for the initiation of rhythmic contractions [90, 91]. Accordingly, the roles of ICC in the regulation of rhythmic contractions are, somehow, limited: when ICC are normally distributed and present, a gastrointestinal organ may still have impaired motility due to electromechanical uncoupling (presence of ICC but lack of contractions); when ICC are damaged or lost, the gastrointestinal organ is expected to have impaired motility. However, it has not been established as to what extent ICC have to be damaged in order for the organ to exhibit impaired motility. The ICC do not directly control the gastrointestinal tone. However, recent *in vitro* studies have suggested that certain subtypes of ICC are involved in the neurotransmission between the enteric neurons and the smooth muscles, and therefore, play important roles in the regulation of the gastrointestinal tone since the tone is largely regulated by the enteric neurons *via* certain neurotransmitters such as nitric oxide (NO). A detailed discussion on the roles of ICC in neurotransmission is presented in the following sections.

### Roles of ICC in regulating peristaltic contractions

A number of studies (mostly *in vivo*) have investigated the roles of ICC in regulating peristaltic contractions along the gut. Impaired gastrointestinal contractions have been frequently reported in all three Kit-mutant models of rats and mice discussed previously [16, 28, 34, 36–41, 92].

In an *in vivo* study performed in Huizinga's lab, movement of barium sulfate in the small intestine of W/W^v^ mice was monitored using radiography. Regular peristaltic waves were observed and distal movement of intestinal contents was noted in the control mice, but was absent in the W/W^v^ mice [75]. The action potentials and contractions appeared random, the contents of the small intestine moved back and forth in an irregular manner in the W/W^v^ mice and the net propulsive effect of the contractile activity in the W/W^v^ mice was much weaker than that in the control mice. However, no difference was observed in the small intestinal transit between control mice and W/W^v^ mice in a study performed in our lab in which movement of a non-nutrient dye (phenol red) through the entire small intestine was assessed quantitatively [78]. Transit studies with various nutrient-rich materials are yet to be performed. The role of ICC in the regulation of intestinal contractions was studied in an *in vivo* study in Ws/Ws rats using strain gauge transducers. Compared to the control rats, the ICC-deficient (a 95% loss of ICC-MY) Ws/Ws rats showed a reduced small intestinal contractile force in the fed state and an absence of regular migrating motor complex in the fasting state. A reduced contractile force and an impaired propagation of contractions in the ileum were reported in an *in vitro* study in W/W^v^ mice [93].

In the colon, impaired colonic contractions were recorded in Ws/Ws rats in which the density of Kit-positive cells was markedly reduced. The wild-type, but not Ws/Ws, rats showed low- and high-frequency cyclic depolarization that was associated with highly regular myogenic motor patterns at the same frequencies. In Ws/Ws rats, irregular patterns of action potentials triggered irregular muscle contractions, occurring within a band width of 10–20 cpm. Spontaneous activity of the nitrergic nerves caused a sustained inhibition of the muscle activity in both wild-type and Ws/Ws rats [28]. In another *in vivo* study, the number of contractions in both the ascending and sigmoid colon in Ws/Ws rats was found to be significantly lower than that in the control rats [94]. An *in vitro* muscle strip study, however, showed unaltered colonic contractions in the proximal colon of Ws/Ws mutant rats [41].

In summary, ample data exist in the literature showing motility abnormalities with the loss of ICC [95], and these findings are consistent with the physiological findings in patients: abnormalities in slow waves lead to impaired motility of the gut [60]. There is no doubt that ICC play an important role in controlling gastrointestinal motility.

## Roles of ICC in mediating neural transmission

While slow waves determine the timing, frequency and propagation of gastrointestinal contractions, and an impairment in the slow waves leads to a disturbed gastrointestinal motility, there is no one-to-one correlation between the slow waves and contractions. The excitation and inhibition of gastrointestinal muscles are achieved by neural inputs from the enteric nerves mainly *via* the cholinergic and nitrergic pathways. Accordingly, a comprehensive control of gastrointestinal motility by ICC cannot be achieved unless ICC are also involved in the transmission of neural transmitters from the enteric nerves to the gut muscles. A number of recent *in vitro* studies have indeed suggested such a pivotal role of ICC in neurotrans-mission. However, conflicting results have been reported in a number of *in vivo* studies, which is the subject of this section.

### *In vitro* studies

A number of *in vitro* studies have suggested that ICC-IM in the stomach and ICC-DMP in the small intestine mediate the enteric neural input to the gastrointestinal muscle cells.

#### LOS and pylorus sphincter

In the LOS and pyloric sphincter, ICC-IM was reported to mediate nitrergic neurotransmission between the enteric nerves and muscles [42]. The LOS and pylorus sphincter contain spindle-shaped ICC-IM that form close relationships with the NO synthase-containing nerve fibers. The pylorus contains ICC within the myenteric plexus and c-Kit immunopositive cells along the submucosal surface of the circular muscle. In W/W^v^ mice, ICC-IM was reported to be absent in the LOS and pylorus, but the distribution of inhibitory nerves was found to be normal. An *in vitro* study showed that NO-dependent inhibitory neurotransmission was reduced and hyperpolarizations to sodium nitroprusside were also attenuated in W/W^v^ mice. The authors concluded that ICC-IM play an important role in NO-dependent neurotransmission in the LOS and pylorus, and the loss of ICC-IM may interfere with relaxations and normal motility in these sphincters [42].

#### Stomach

Similar to the LOS and pylorus sphincter, there is a loss of ICC-IM but normal distribution of inhibitory nerves in the stomach. However, the NO-dependent inhibitory neuroregulation was reported to be greatly reduced, and smooth muscle tissues relaxed in response to exogenous sodium nitroprusside, whereas the membrane potential effects of sodium nitroprusside were attenuated [26]. These data suggested that ICC-IM in the stomach play a critical role in NO-dependent neurotransmission in the stomach. A similar role of ICC-IM in cholinergic excitatory neurotransmission was also reported [96]: in W/W^v^ mice, there was a loss of neural responses in the smooth muscles to cholinergic stimulation [97].

#### Small intestine

In the small intestine, ICC-IM are replaced by a dense network of ICC located at the level of deep muscular plexus; ICC-DMP are intimately associated with the enteric nerve terminals [98]. The enteric nerve terminals appear to form synapses preferentially with ICC-DMP rather than the smooth muscle cells [99]. In an ultrastructure study, it was found that ICC-DMP were innervated by both cholinergic and nitrergic nerves and were the only cells to possess specialized synapse-like junctions with nerve varicosities and gap junction contacts with the smooth muscle cells [100]. The functional role of ICC-DMP is difficult to prove since they persist in the small intestine of W/W^v^ and Sl/Sl^d^ mutants. However, the loss of ICC-DMP by blocking Kit was shown to cause loss of cholinergic and nitrergic neural responses [101], suggesting that in the small intestine, ICC-DMP play a critical role in the cholinergic and nitrergic neurotransmission.

#### Colon

In the colon, the findings of an *in vitro* study do not seem to support the role of ICC in the nitrergic neurotransmission [28]. In Ws/Ws rats, all subtypes of ICC were found to be lost by more than 90% in the middle and distal colon. Compared to the wild-type, the muscle strips of Ws/Ws rats showed irregular muscle contractions. However, spontaneous activity of nitrergic nerves caused a sustained inhibition of the muscle activity in both wild-type and Ws/Ws rats. Electrical field stimulation of enteric nerves, after blockade of cholinergic and adrenergic activity, elicited inhibition of the mechanical activity and biphasic inhibitory junction potentials in both wild-type and Ws/Ws rats [28]. These data suggest that ICC are not essential for the nitrergic neural transmission.

### *In vivo* studies

On the contrary, most of the *in vivo* studies do not seem to support the theory that ICC-IM or ICC-DMP are critical for neuro-transmission. The findings of these *in vivo* studies are discussed in this section.

#### Lower oesophageal sphincter

In the LOS, ICC-IM do not seem to play a role in the nitrergic neurotransmission. The LOS mains a basal tone and relaxes with swallowing that is caused by the activation of inhibitory nonadrenerignoncholingergic nerves. NO has been shown to be a major inhibitory neurotransmitter. In mice with neuronal NO synthase gene disruption (*nNOS*^−/–^), W/W^v^ mice lacking ICC-IM and control mice, an intraluminal manometry was performed under anaesthesia [102]. The LOS in the nNOS^−/–^ mice was significantly hypertensive and its relaxation to swallowing and efferent vagal stimulation was markedly impaired. In contract, the LOS in the W/W^v^ mice is hypotensive and relaxes normally to swallowing and efferent vagal activation. Similar normal relaxation to electrical stimulation was also noted in the LOS circular muscle strip of W/W^v^ mice [103], suggesting that despite the absence of c-Kit-positive ICC, a nerve–muscle interaction can be accomplished likely by the diffusion of neurotransmitters to the smooth muscle cells.

#### Stomach

The role of ICC-IM in the mediation of neurotransmission in the stomach was denied in a recent *in vivo* study investigating gastric adaptive relaxation in W/W^v^ mice [36]. Gastric accommodation refers to an *in vivo* reflex that causes relaxation of the stomach to avoid undue intraluminal pressure due to filling of the stomach. This reflex involves the vagal and intrinsic enteric nerves stimulated by the distention of the stomach. Upon gastric distention, the vagal nerves are activated and an inhibitory neurotransmitter, NO, is released and the stomach is relaxed. In the recent *in vivo* study, gastric accommodation was assessed in the isolated whole stomach of wild-type and W/W^v^ mice [103]. It was reported that despite the absence of ICC-IM, normal gastric adaptive relaxation occurred in the W/W^v^ mice stomach, suggesting that ICC-IM are not essential for the activation of NO-mediated relaxation. Similar to the *in vivo* study of the LOS [102], the basal tone of the stomach in the W/W^v^ mice was found to be lower than the control mice. The discrepancy in the findings between the *in vitro* and *in vivo* studies is explained in a recent study [104]. In a carefully designed *in vitro* study, Huizinga *et al.* examined the responses of muscle strips taken from the fundus of Ws/Ws rats to electrical field stimulation and found that the lack of inhibitory responses to the electrical field stimulation was not due to the inability of neurotransmissions to reach the smooth muscle cells but rather due to the lack of an active tone, which was also reported in the LOS [102]. In addition, nerve stimulation excited the noncholinergic excitatory nerves that masked the effect of the inhibitory nerves [104].

#### Colon

Peristaltic reflex is a well-known phenomenon in the small intestine and colon, mediated by the cholinergic excitation and nitrergic relaxation. One *in vivo* study in W/W^v^ mice has demonstrated that ICC may have no important role in this neural peristaltic reflex [45]. In the intestine, local distention by either inflation of a small balloon or mechanical distention induces contraction (ascending contraction) and relaxation (descending relaxation) at the regions oral and anal to the distended region, respectively. Such neural reflexes are of great importance since they move the intestinal bolus from the proximal gut to the distal gut. The ascending contraction is mediated by the cholinger-gic or tachykinin pathway, whereas the descending relaxation is achieved *via* the nitrergic mechanism. The segment of distal colon taken from the W/W^v^ mice showed a normal ascending contraction and a descending contraction in response to balloon distention of the segment [45].

#### Anal sphincter

In the anal sphincter, *in vivo* studies reported similar findings as in the stomach and colon, suggesting limited or no roles of ICC in the nitrergic relaxation of the anal sphincter [17, 105]. In one *in vivo* study, the anal sphincter of W/W^v^ mice showed a reduced basal pressure and normal relaxation to rectal distention, a phenomenon called rectoanal inhibitory reflex (RAIR) mediated by the inhibitory nitrergic pathway [105]. In another *in vivo* study, distention of the rectum elicited a volume-dependent relaxation of the anal sphincter in W/W^v^ mice; the degree of relaxation was lower than that in the control mice at low distention volume but comparable to the control nice at high distention volume [17]. The *in vitro* experiment of the same study also failed to confirm the role of ICC-IM in neurotransmission. Electrical stimulation of the internal anal sphincter of W/W^v^ mice relaxed to the same extent as those from controls. In addition, blockade of NO biosynthesis greatly reduced these relaxations, indicating that nitrergic innervation was intact. The latter was further confirmed by immunohistochemical staining. Since ICC-IM was lacking in the W/W^v^ mice, these results argue against a role for ICC as an intermediate between the nitrergic nerves and smooth muscle cells [17].

## Roles of ICC as mechanoreceptors

**Fig. 4:**
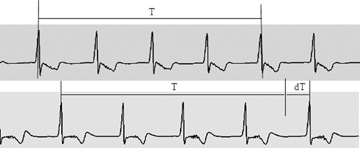
Effects of gastric distention on gastric slow wave frequencies in a dog. Top: 80-sec. slow wave recordings from a pair of serosal electrodes at the baseline. Bottom: 80-sec. slow wave recordings from the same pair of serosal electrodes when the stomach was distended by an intragastric balloon with a pressure of 40 mmHg. An obvious decrease in slow wave frequency can be appreciated by comparing the time intervals covering four cycles of slow waves at the baseline (T) and during distention (T + dT): the longer time period for the four slow waves during distention is indicative of a decrease in the slow wave frequency.

The theory that ICC act as mechanoreceptors in the gut is based on morphological studies and one single physiological study [29, 106, 107]. Based on an *in vitro* study, it was theorized as follows: the ingestion of a meal stretches the antrum; the ICC-IM in the antrum sense this stretch, and the sensing mechanisms in the ICC-IM transiently depolarize the smooth muscle cells to cause an increase in the slow wave frequency and a decrease in its amplitude. This hypothesis is based on the finding that the antral muscle strips from W/W^v^ mice lacking ICC-IM do not exhibit the transient increase in the slow wave frequency and decrease in its amplitude [29]. While not much information is available in the literature to support or dispute the above hypothesis, *in vivo* physiological data seem to be against the notion that the gastric slow wave frequency increases transiently after the ingestion of a meal or when the stomach is distended. A number of *in vivo* physiological studies have actually shown the opposite: there is a transient decrease in the slow wave frequency upon food ingestion, measured by serosal bipolar electrodes as well as cutaneous abdominal electrodes, a method called electrogastrography [46, 108–111]. In an *in vivo* canine study with the gastric slow waves measured from a pair of electrodes placed on the gastric serosa, it was found that the frequency of the gastric slow waves decreased linearly with greater initial meal volume. A transient frequency dip in the gastric slow waves after a meal has been frequently and consistently reported in electrogastrographic studies [109–114]. Figure 4 shows a typical decrease in the slow wave frequency measured in a conscious dog using a pair of gastric serosal electrodes when the stomach was distended by an intra-gastric balloon; the frequency of the gastric slow wave was about 6 cpm before gastric distention and reduced to about 5 cpm during gastric distention.

However, there is lack of *in vivo* studies to test the hypothesis that ICC serve as mechanoreceptors.

## Conclusions

To better understand the roles of ICC in regulating gastrointestinal motility, the *in vitro* and *in vivo* physiological motility studies and their findings are discussed in this article. Based on morphological and in vitrophysiological studies, ICC are believed to be pacemaker cells generating slow waves to serve as mediators in neural transmission and may also act as mechanoreceptors.

The theory that ICC-MY are pacemaker cells seems well established based on numerous *in vitro* studies. However, pacemaker activity as observed *in vivo* may be complex and involve smooth muscle cells. (1) A recent *in vivo* study demonstrated the presence of slow waves in the small intestine of W/W^v^ mice with the absence of ICC-MY, (2) some *in vitro* studies also showed rhythmic electrical activities called action potentials and suggested that these electrical activities may be generated by smooth muscles and (3) although motor abnormalities *in vivo* are associated with the loss of ICC, further *in vivo* evidence is needed.

The role of ICC-IM or ICC-DMP in mediating neural transmission between the enteric nerves and the gut muscles is established according to the majority of *in vitro* studies. However, findings *in vivo* motility studies do not seem to support the role of ICC-IM as neural mediators, and recent *in vitro* studies have demonstrated alternative interpretations of previous findings. Accordingly, one has to exercise caution in interpreting *in vitro* and *in vivo* data regarding the role of ICC-IM or ICC-DMP in neural transmission.

The hypothesis that ICC-IM may also act as mechanoreceptors needs further investigation. However, in vivo physiological findings thus far have not provided evidence.

The clinical significance of ICC is dependent on the roles of ICC in regulating gastrointestinal motility. There is no doubt that the damage or loss of ICC results in dysmotility in the gut. However, future studies are needed to establish a quantitative correlation between the loss of ICC and the severity of motility disorders. If this can be established, ICC may be used as biomarkers of gastrointestinal motility disorders. More studies are needed to confirm the role of ICC as mediators of neurotransmission. If ICC are truly involved in neurotransmission, they will be of great clinical significance.
